# Dietary supplementation with branched-chain amino acids enhances milk production by lactating sows and the growth of suckling piglets

**DOI:** 10.1186/s40104-022-00718-y

**Published:** 2022-06-17

**Authors:** Reza Rezaei, Ana San Gabriel, Guoyao Wu

**Affiliations:** 1 Department of Animal Science, Texas A&M University, College Station, TX 77843 USA; 2 Ajinomoto Co., Inc, 1-15-1 Kyobashi, Chuoku, Tokyo, 104-8315 Japan

**Keywords:** Branched-chain amino acids, Lactation, Milk synthesis, Milk yield, Neonatal growth, Sow

## Abstract

**Background:**

Under current dietary regimens, milk production by lactating sows is insufficient to sustain the maximal growth of their piglets. As precursors of glutamate and glutamine as well as substrates and activators of protein synthesis, branched-chain amino acids (BCAAs) have great potential for enhancing milk production by sows.

**Methods:**

Thirty multiparous sows were assigned randomly into one of three groups: control (a corn- and soybean meal-based diet), the basal diet + 1.535% BCAAs; and the basal diet + 3.07% BCAAs. The ratio (g/g) among the supplemental L-isoleucine, L-leucine and L-valine was 1.00:2.56:1.23. Diets were made isonitrogenous by the addition of appropriate amounts of L-alanine. Lactating sows had free access to drinking water and their respective diets. The number of live-born piglets was standardized to 9 per sow at d 0 of lactation (the day of parturition). On d 3, 15 and 29 of lactation, body weights and milk consumption of piglets were measured, and blood samples were obtained from sows and piglets 2 h and 1 h after feeding and nursing, respectively.

**Results:**

Feed intake did not differ among the three groups of sows. Concentrations of asparagine, glutamate, glutamine, citrulline, arginine, proline,  BCAAs, and many other amino acids  were greater (*P* < 0.05) in the plasma of BCAA-supplemented sows and their piglets than those in the control group. Compared with the control, dietary supplementation with 1.535% and 3.07% BCAAs increased (*P* < 0.05) concentrations of free and protein-bound BCAAs, glutamate plus glutamine, aspartate plus asparagine, and many other amino acids in milk; milk production by 14% and 21%, respectively; daily weight gains of piglets by 19% and 28%, respectively, while reducing preweaning mortality rates by 50% and 70%, respectively.

**Conclusion:**

Dietary supplementation with up to 3.07% BCAAs enhanced milk production by lactating sows, and the growth and survival of their piglets.

## Introduction

The major goal of the swine industry is to minimize neonatal mortality and improve weaning weights of neonatal pigs because their adaptability to weaning and their rates of growth to market weight are positively affected by weaning weight [1]. The total net profit of a swine production unit is primarily determined by both the number and size of piglets produced per year per sow [2]. Recent attempts to increase litter size have led to increased prevalence of low-birth-weight piglets [3, 4], which results in a higher rate of mortality and a longer period of time to reach an optimum market weight, when compared with normal-birth-weight piglets [5, 6]. Pork quality is also affected by birth weight to a large extent because of alterations in muscle metabolism [3]. The greatest proportion of mortality in commercial swine production occurs prior to weaning and ranges between 10% and 20% of live-born pigs [4, 7]. For example, the rates of pre-weaning mortality of piglets in U.S. commercial swine herds were 15.0% for the mean live-born litter size of 9.4 in 1992 [8] and 15.4% for the mean live-born litter size of 13.5 in 2020 [9] due to multiple factors including low birth weight, the inadequate provision of milk, intestinal dysfunction, physical (particularly muscular) weakness, and crushing by the sow [1, 10].

Insufficient intake of dietary amino acids (AAs) limits milk production by lactating sows [11, 12]. For example, increasing the content of crude protein (CP; 23% vs. 16.2%) and all proteinogenic AAs, including branched-chain AAs (BCAAs; e.g., 4.61% vs. 2.28%) in the diet of lactating sows enhances milk yield and piglet growth [13]. It is unknown whether this effect is due to specific AAs or simply total AA nitrogen. Results from several studies indicate that dietary supplementation with valine [14–17] or isoleucine [18], but not leucine [16], increase the litter weight of suckling piglets. BCAAs are major AA components of milk protein [19, 20], and also provide the amino group for the synthesis of glutamate, glutamine, aspartate and asparagine [21], all of which are abundant AAs in milk [22]. To date, dietary supplementation with a single BCAA to sows has yielded inconsistent results on milk production [14–16] possibly due to an imbalance of the three BCAAs in diets [23]. For example, dietary supplementation of lactating sows with 0.4% isoleucine enhanced the growth of suckling piglets when the ratio of isoleucine to leucine in the complete diet was 0.88 [14], but isoleucine failed to exert a beneficial effect when the ratio of isoleucine to leucine in the complete diet was 0.55 [16]. By contrast, increasing the ratio of total valine to total lysine in the diet (containing 0.71% standardized ileal digestible lysine) of the lactating sow nursing 14 piglets from 0.84 to 0.91 did not affect the preweaning mortality and average daily weight gain of the litter, litter weight at weaning, average milk yield, and the concentrations of fat, protein and lactose in milk [24]. Currently there is no information about the effects of dietary supplementation with leucine plus isoleucine plus valine versus an isonitrogenous control on lactation performance in any mammals (including sows).

L-Leucine activates the mechanistic target of rapamycin (MTOR) cell signaling pathway to stimulate protein synthesis in skeletal muscle [24–26] and mammary epithelial cells [27]. We recently reported that physiological concentrations of L-valine also stimulated the MTOR signaling and protein synthesis but inhibited proteolysis in porcine mammary epithelial cells [28]. We hypothesized that a proper mixture of BCAAs would increase milk synthesis in the mammary tissue of lactating sows, thereby improving neonatal growth, development and survival. The present study tested this hypothesis by determining effects of BCAA supplementation to lactating sows on: the preweaning growth and survival of the litter, the yield and nutrient composition of milk, and the concentrations of biochemical metabolites in the plasma of sows and piglets.

## Materials and methods

### Animals housing and management for feeding trials

The experimental protocol for this study was approved by the Texas A&M University Institutional Animal Care and Use Committee. Thirty multiparous sows (Yorkshire × Landrace; 12 sows at parity 2 (approximately 16 months of age and an average BW of 152 kg) and 18 sows at parity 3 (approximately 22 months of age and an average BW of 173 kg)) were housed individually in 1.8 m × 2.5 m pens with plastic coated perforated flooring. During the entire gestation, they were fed 2 kg/d of a corn- and soybean meal-based diet containing 12.0% CP and 3083 kcal/kg metabolizable energy [29]. Immediately after farrowing (d 0), the sows were assigned randomly to one of three groups, and fed a corn- and soybean meal-based diet (Table 1) supplemented with 0 (control), 1.535% (0.32% L-isoleucine plus 0.82% L-leucine plus 0.395% L-valine), or 3.07% BCAAs (0.64% L-isoleucine plus 1.64% L-leucine plus 0.79% L-valine). The basal diet contained 3.20% total BCAAs, and its nutrient composition was analyzed as we described [20]. The content of AAs in all the three formulated experimental diets was determined and verified as we described [20] before feeding to sows. There were 10 sows per treatment group (4 sows at parity 2 and 6 sows at parity 3). Diets were provided to sows twice a day (3 kg/meal; 6 kg/d) and they had free access to drinking water. No sow consumed all the 6-kg diet on each day of lactation. The supplemental doses of BCAAs were based on the following rationale to prevent an imbalance among BCAAs. First, the ratios of isoleucine:leucine:valine in the lumen of the jejunum of lactating sows fed a typical corn- and soybean meal-based diet were determined to be 1.00:2.56:1.23 [30]. The concentrations of BCAAs in the lumen of the jejunum (the main site of AA absorption) result from the balance among dietary AA intake, intestinal AA secretion, and intestinal microbial metabolism. We considered that these ratios of BCAAs would not affect their absorption by the enterocytes of the small intestine. Second, the content of leucine in the basal diet was 1.64%. Thus, the supplemental doses of 0.82% and 1.64% leucine amounted to 50% and 100% of the leucine content in the basal diet. Diets were made isonitrogenous and isoenergetic by the addition of appropriate amounts of L-alanine at the expense of corn starch (Table 2). Supplemental AAs were obtained from Ajinomoto Co., Inc. (Tokyo, Japan). Before feeding sows, complete diets were mixed thoroughly using a Hobart feed mixer.

**Table 1:** Composition of the basal diet for lactating sows on an as-fed basis

Item	Basal diet^1^, %
Ingredient	
Corn grain	57.50
Soybean meal (44.5% CP)	27.00
Cornstarch	3.07
Sugarcane molasses	2.78
Potassium chloride	0.10
Salt	0.35
Vitamin-mineral premix^2^	3.00
Vegetable oil	3.00
Dicalcium phosphate	2.50
Limestone	0.70
Analyzed chemical composition	
DM, %	90.0
ME, Mcal/kg	3.32
CP^3^, %	17.5
Ca, %	1.04
Available P, %	0.54
Total P, %	0.79

^1^This basal lactation diet was used to prepare experimental diets for sows  from farrowing to d 29 of lactation

^2^The vitamin premix provided the following per kilogram of complete diet (as-fed basis): 46.7 mg of Mn as manganous oxide; 75 mg of Fe as iron sulfate; 103.8 mg of Zn as zinc oxide; 9.5 mg of Cu as copper sulfate; 0.72 mg of I as ethylenediamine dihydroiodide; 0.23 mg of Se as sodium selenite; 7556 IU of vitamin A as vitamin A acetate; 825 IU of vitamin D_3_; 61.9 IU of vitamin E; 4.4 IU of vitamin K as menadione sodium bisulfate; 54.9 μg of vitamin B_12_; 13.7 mg of riboflavin; 43.9 mg of D-pantothenic acid as calcium pantothenate; 54.9 mg of niacin; and 1650 mg of choline as choline chloride

^3^The basal diet provided the following amino acids (%, as-fed basis): alanine, 0.94; arginine, 1.09; asparagine, 0.79; aspartate, 1.12; cysteine, 0.30; glutamine, 1.68; glutamate, 1.56; glycine, 0.86; histidine, 0.44; isoleucine, 0.74; leucine, 1.64; lysine, 0.90; methionine, 0.28; phenylalanine, 0.87; proline, 1.45; serine, 0.84; threonine, 0.65; tryptophan, 0.21; tyrosine, 0.71; and valine, 0.82

**Table 2:** Supplemental branched-chain amino acids (BCAAs) and L-alanine (the isonitrogenous control) to the basal corn-soybean diet^1^

Treatment	Supplemental amino acid, g/100 g diet				Total nitrogen, mmol N/100 g diet
	L-Isoleucine	L-Leucine	L-Valine	L-Alanine	
Control	0.00	0.00	0.00	2.149	24.12
1.535% BCAA	0.32	0.82	0.395	1.07	24.12
3.07% BCAA	0.64	1.64	0.79	0.00	24.12

^1^The diets were made isonitrogenous with the addition of L-alanine at the expense of corn starch

On the day of farrowing (d = 0), cross fostering of newborn pigs was performed to minimize within-litter variation, and the number of piglets per sow was standardized to nine, yielding a total of 270 piglets for this experiment. The number of male and female piglets per treatment group was the same. The ambient temperature in the farrowing facility was maintained at 26 °C throughout the trial. Heat lamps were used to provide heat to neonatal pigs in each pen. Feed intake of the sows was recorded daily. Teeth of piglets were clipped and their tails docked at d 3 of age. At this age, each piglet was also ear notched for identification and given an intramuscular iron dextran injection (200 mg/piglet). On d 0, 3, 15 and 29 of lactation, all sows and piglets were weighed. We did not obtain the baseline information on metabolites in milk as well as maternal and neonatal plasma on d 0 of lactation to avoid possible negative effects of milk- and blood- sampling procedures on sows and newborn pigs. During the entire experiment, piglets had no access to the feed provided to sows and were not provided with any creep feed or milk replacer. The growth performance and survival rates of piglets were determined.

### Collection of blood samples from sows and piglets

Heparinized tubes were used to draw blood from sows and piglets on d 3, 15, and 29 of lactation. Specifically, blood samples (10 mL) were obtained from all sows via the caudal auricular vein in the ear 2 h post-feeding, and blood samples (2 mL) were obtained from two randomly selected piglets in each litter via the jugular vein 1 h after nursing. Blood samples were immediately centrifuged at 10,000 × *g* for 1 min, and the supernatant fluid (plasma) stored at − 80 °C for subsequent analyses.

### Collection of milk from sows

To obtain milk samples, 20 IU oxytocin was administered via the ear vein of sows to induce milk letdown into the teat canal and milk was expressed manually [20]. Milk samples (50 mL/gland) were collected from three different mammary glands (front, middle, and rear). Equal volumes of milk (10 mL) from each gland of a sow were mixed, and the samples stored at − 80 °C for subsequent analyses.

### Milk consumption of piglets

Milk consumption of piglets was determined between 10:00 (the initial isolation of piglets from sows) and 20:00 (the last measurement) using the weigh-suckle-weigh method as previously described [31]. Briefly, on d 3, 15, and 29 of lactation, after blood sampling, piglets were separated from their mothers every 2 h and then returned to the sows for 1-h nursing. Piglets in the same litter were weighed as a group before and after nursing to calculate milk intake. This same procedure was performed five times and applied to all litters.

### Proximate analysis of milk composition

The frozen whole-milk samples were thawed at 4 °C, and then used for determination of total milk fat, CP, dry matter (DM), and ash according to AOAC (Association of Official Analytical Chemists, Washington DC) procedures [32]. Additionally, protein was determined by the modified Lowry procedure using purified porcine immunoglobulin G (IgG) as the standard [20]. Milk lactose was determined using the colorimetric method of Marier and Boulet [33] that involved the reaction with phenol and sulfuric acid and then reading against the blank at 490 nm. Total milk lipids were extracted using a mixture of isopropylalcohol:heptane:0.5 mol/L H_2_SO_4_ [34].

### Analysis of free AAs in plasma

Plasma samples (0.5 mL) were deproteinized with an equal volume of 1.5 mol/L HClO_4_ and neutralized with 0.25 mL of 2 mol/L K_2_CO_3_. The neutralized samples were analyzed for free AAs by HPLC methods involving precolumn derivatization with *o*-phthaldialdehyde [35].

### Analysis of free and protein-bound AAs in milk

For analysis of free AAs, milk samples (0.5 mL) were deproteinized with an equal volume of 1.5 mol/L HClO_4_ and neutralized with 0.25 mL of 2 mol/L K_2_CO_3_. The neutralized samples were analyzed for free AAs, as described above. Composition of protein-bound AAs in milk was determined as described by Dai et al. [36].

### Analysis of glucose, ammonia, urea, lactate, pyruvate and ketone bodies in plasma and milk

Deproteinized and neutralized plasma or milk samples were analyzed for glucose, ammonia, urea, lactate, pyruvate, and ketone bodies by enzymatic methods [37, 38]. The enzymes were hexokinase, glucose-6-phosphate dehydrogenase, glutamate dehydrogenase, urease, lactate dehydrogenase, and β-hydroxybutyrate dehydrogenase for the assays of glucose, ammonia plus urea, lactate plus pyruvate, acetoacetate plus β-hydroxybutyrate, respectively.

### Determination of plasma thiobarbituric acid reactive substances (TBARS)

To evaluate the oxidative status of sows and piglets, their plasma was analyzed for TBARS. Thiobarbituric acid forms a reaction with malondialdehyde (a naturally occurring product of lipid peroxidation) under high temperature and acidic conditions and can be measured colorimetrically [39]. The technical procedure was performed as recommended by the manufacturer (Cayman Chemical, Ann Arbor, MI, USA). Briefly, 100 μL of thawed plasma was placed into a labeled glass tube and mixed with the reagents of the commercial kit (Cayman Chemical, Cat # 10009055). Each tube was capped and incubated at 95 °C for 60 min. The tubes were then cooled in an ice bath for 10 min. Thereafter, the tubes were centrifuged at 1100 × *g* for 15 min and the supernatant fluid was obtained for the measurement absorbance at 530–540 nm.

### Statistical analysis

Results are expressed as means ± pooled SEM. Data were statistically analyzed using two-way ANOVA and the Student-Newman-Keuls multiple comparison, as we described [40]. The two factors were treatment (BCAA supplementation) and lactation day. Our web-based statistical program is freely available online at https://houssein-assaad.shinyapps.io/TwoWayANOVA/. Data on the rates of piglet mortality were analyzed by *X*^2^-analysis [41]. Values of *P* ≤ 0.05 are taken to indicate statistical significance.

## Results

### Feed intake and body weights of lactating sows

There were no differences (*P* > 0.05) in feed intake by sows among groups, which was approximately 32 g/kg BW per day throughout the 29-d period of lactation (Table 3). With advanced lactation, sows mobilized body reserves to provide nutrients for milk production. On the day of farrowing, body weights of sows did not differ (*P* > 0.05) among the three treatment groups (Table 4). However, on d 29 of lactation, sows in the 3.07% BCAA group were heavier (*P* < 0.05) than those in the control and 1.535% BCAA groups (Table 4). Overall, sows in the control and 1.535% BCAA groups lost (*P* < 0.05) 26 and 16 kg body weight between d 0 and 29 of lactation, respectively, whereas there was no difference (*P* > 0.05) in the body weights of sows in the 3.07% BCAA group during the same period (Table 4). Most (approximately 60%) of the weight losses in sows occurred during the fourth week of lactation.

**Table 3:** Effects of supplementing branched-chain amino acids (BCAAs) to the diets of sows on their feed intake and the milk consumption of piglets during lactation

Variable	Control			1.535% BCAA			3.07% BCAA			Pooled SEM	***P***-value		
	d 3	d 15	d 29	d 3	d 15	d 29	d 3	d 15	d 29		Treatment	Day	T × D^**1**^
Milk consumption by piglets, mL/kg BW/d	284	231	153	315	252	178	342	277	193	6.4	< 0.01	< 0.01	0.291
	**d 0**–**15**	**d 15**–**29**	**d 0**–**29**	**d 0**–**15**	**d 15**–**29**	**d 0**–**29**	**d 0**–**15**	**d 15**–**29**	**d 0**–**29**				
ADFI^2^ by sows, g/kg BW/d	29.7	34.2	32.0	29.9	33.7	31.8	29.7	34.0	31.9	3.1	0.997	0.069	1.00

Values are means with pooled SEM, *n* = 10 sows/treatment group. See Table 6 for the number of piglets on d 3, 15 and 29 of lactation.

Data were analyzed by two-way ANOVA.

^1^Treatment (T), Day (D), and Treatment × Day (T × D) interaction

^2^*ADFI* Average daily feed intake

**Table 4:** Effects of supplementing branched-chain amino acids (BCAAs) to the diets of sows on body weights of sows and piglets as well as their weight changes during lactation

Variable	Control				1.535% BCAA				3.07% BCAA				Pooled SEM	***P***-value		
	d 0	d 3	d 15	d 29	d 0	d 3	d 15	d 29	d 0	d 3	d 15	d 29		Treatment	Day	T × D^**1**^
Sow BW, kg	187	184	173	161	185	182	175	169	187	185	183	181	9.1	0.198	0.01	0.791
Piglet BW, kg	1.40	1.84	4.34	9.54	1.41	1.94	5.05	11.5	1.42	2.03	5.34	12.9	0.10	< 0.001	< 0.01	< 0.01
	**d 0–3**	**d 3–15**	**d 15–29**	**d 0–29**	**d 0–3**	**d 3–15**	**d 15–29**	**d 0–29**	**d 0–3**	**d 3–15**	**d 15–29**	**d 0–29**				
ADG of piglets, g/d	147	208	371	281	177	259	460	348	203	276	540	396	6.7	< 0.001	< 0.01	< 0.01
BW loss of sows, kg	3.4	11.1	11.7	26.2	3.3	7.4	6.0	16.7	1.3	2.5	2.2	6.0	0.98	< 0.001	< 0.01	< 0.01

Values are means with pooled SEM, *n* = 10 sows/treatment group. See Table 5 for the number of piglets on d 3, 15 and 29 of lactation.

Data were analyzed by two-way ANOVA.

^1^Treatment (T), Day (D), and Treatment × Day (T × D) interaction

### Milk intake, growth and mortality of sow-reared piglets

Milk intake of piglets was 16.5% and 20.5% greater (*P* < 0.05) in the 1.535% and 3.07% BCAA groups, respectively, compared with control piglets (Table 3). Piglet BW at d 0 of lactation did not differ (*P* > 0.05) among treatment groups (Table 4). However, the BW of piglets nursed by sows fed the BCAA-supplemented diets during lactation were greater (*P* < 0.01) at d 15 and 29 of lactation, compared with piglets from control-fed sows. Average daily weight gains of piglets from sows fed the BCAA-supplemented diets during lactation were consistently greater (*P* < 0.01) throughout the 29-d lactation period, compared with piglets from control-fed sows (Table 4). Piglets in the 3.07% BCAA group had a greater weight gain (*P* < 0.01) than piglets in the 1.535% BCAA group. Of note, the rates of preweaning mortality of piglets in the 1.535% and 3.07% BCAA groups were 50% and 33% (*P* < 0.05) of the value for the control group, respectively (Table 5).

**Table 5:** Effects of supplementing branched-chain amino acids (BCAAs) to the diets of sows on the rates of mortality of sow-reared piglets during lactation

Group	Number of piglets				Rate of mortality, %			
	d 0	d 3	d 15	d 29	d 0^**1**^	d 0–3	d 0–15	d 0–29
**Control**	90	78	77	76	0	13.3	14.4	15.6
**1.535% BCAA**	90	84	84	84	0	6.7	6.7	6.7
**3.07% BCAA**	90	86	86	86	0	4.4	4.4	4.4

Values are the numbers of live piglets

Data were analyzed by *X*^2^-analysis, which revealed that BCAA supplementation reduced the rate of preweaning mortality (*P* = 0.021) as compared to control

^1^Time of assignment to treatment groups

### Milk composition (proximate analysis)

Concentrations of DM, CP, fat, carbohydrates (including lactose), and minerals were higher (*P* < 0.01) in milk from BCAA-supplemented sows as compared to control sows (Table 6). Lactose accounted for 89.3% to 89.8% of total carbohydrate in each group. Composition of milk did not differ (*P* > 0.05) between the 1.535% and 3.07% BCAA groups. Concentrations of DM, fat and CP in milk were greater (*P* < 0.05) on d 3 than on d 15 or d 29 of lactation, but did not differ (*P* > 0.05) between d 15 and 29 of lactation. There was no treatment × day interaction effect (*P* > 0.05) on milk composition based on proximate analysis (Table 6).

**Table 6:** Effects of supplementing branched-chain amino acids (BCAAs) to the diets of sows on the proximate composition of their milk during lactation

Variable	Control			1.535% BCAA			3.07% BCAA			Pooled SEM	***P***-value		
	d 3	d 15	d 29	d 3	d 15	d 29	d 3	d 15	d 29		Treatment	Day	T × D^**1**^
DM, %	20.8	19.6	19.4	22.4	20.7	20.5	22.8	21.1	21.0	0.478	< 0.01	0.023	0.983
CP, %	5.72	4.93	4.78	6.10	5.16	5.06	6.24	5.19	5.10	0.193	< 0.01	0.011	0.985
Fat, %	8.63	8.04	8.12	9.12	8.44	8.48	9.26	8.66	8.74	0.245	< 0.01	0.002	0.952
Lactose, %	4.99	5.16	5.10	5.61	5.59	5.39	5.71	5.66	5.57	0.206	< 0.01	0.329	0.996
Carb, %	5.58	5.76	5.62	6.24	6.16	6.02	6.37	6.32	6.22	0.206	< 0.01	0.329	0.996
Ash, %	0.87	0.87	0.88	0.94	0.94	0.94	0.93	0.93	0.94	0.025	< 0.01	0.884	1.00

Values are means with pooled SEM, *n* = 10 sows/treatment group

Data were analyzed by two-way ANOVA

^1^Treatment (T), Day (D), and Treatment × Day (T × D) interaction

*Carb* carbohydrate (including lactose)

### Concentrations of AAs in the plasma of sows and piglets 

Concentrations of AAs in plasma of sows and piglets at d 3, 15 and 29 of lactation are summarized in Tables 7 and 8, respectively. BCAA supplementation to sows resulted in greater (*P* < 0.001) concentrations of Pro, BCAAs, citrulline, Arg, Asp, Glu and Gln in plasma, compared with values for sows in the control group. Except for Asn, β-Ala and Lys, concentrations of all other AAs in the plasma from sows were greater (*P* < 0.05) for the 3.07% BCAA group than the 1.535% BCAA group (Table 7). The effect of day of lactation was significant (*P* < 0.05) for all AAs in plasma from sows except for Cys, Glu, Gln, Gly, Lys, Met, Pro and Tau. However, no treatment × day effect was detected for any AAs except Asn in the plasma of sows. Similar results were obtained for concentrations of AAs in plasma from piglets (Table 8).

**Table 7:** Effects of supplementing branched-chain amino acids (BCAAs) to the diets of sows on the concentrations of free amino acids and thiobarbituric acid reactive substances (TBARS) in their plasma during lactation

Variable	Control			1.535% BCAA			3.07% BCAA			Pooled SEM	***P***-value		
	d 3	d 15	d 29	d 3	d 15	d 29	d 3	d 15	d 29		Treatment	Day	T × D^**1**^
Ala	369	492	517	482	600	607	596	640	649	42	< 0.001	< 0.001	0.485
Arg	103	121	150	147	173	177	161	181	182	15	< 0.001	0.001	0.632
Asn	58	63	80	83	85	85	96	92	95	5.9	< 0.001	0.052	0.037
Asp	32	34	36	34	33	36	35	34	38	2.0	0.353	0.005	0.789
β-Ala	17	18	19	18	18	19	18	19	19	0.59	0.021	0.038	0.056
Cit	60	64	74	70	78	90	86	85	91	6.7	< 0.001	0.004	0.597
Cys^2^	229	235	236	231	235	239	243	252	255	14	0.100	0.555	0.999
Gln	564	567	572	620	628	632	654	664	670	26	< 0.001	0.717	1.00
Glu	84	72	80	113	121	125	132	140	143	18	< 0.001	0.494	0.461
Gly	947	964	1003	949	981	1018	1029	981	1023	54	0.429	0.343	0.822
His	76	69	80	80	75	78	91	79	80	4.2	0.005	0.008	0.161
Ile	70	91	114	130	139	146	169	195	219	14	< 0.001	< 0.001	0.545
Leu	148	167	210	260	286	295	341	397	403	30	< 0.001	0.011	0.831
Lys	124	126	130	138	144	147	156	164	163	8.1	< 0.001	0.265	0.984
Met	37	35	34	41	41	41	41	42	50	3.0	< 0.001	0.354	0.083
Orn	69	76	102	76	77	101	78	77	105	6.1	0.465	< 0.001	0.882
Phe	72	77	96	73	73	95	79	73	97	4.7	0.684	< 0.001	0.66
Pro	237	246	247	300	306	320	358	376	385	17	< 0.001	0.162	0.945
Ser	136	137	147	138	141	151	139	142	154	9.1	0.675	0.030	0.997
Tau	55	61	58	58	62	61	58	66	67	5.1	0.149	0.119	0.921
Thr	101	102	127	108	101	132	109	119	132	9.4	0.151	< 0.001	0.692
Trp	49	49	52	53	51	57	54	60	64	3.8	< 0.001	0.038	0.482
Tyr	58	74	91	79	90	92	82	91	99	6.3	< 0.001	< 0.001	0.194
Val	182	216	234	276	326	357	374	416	473	32	< 0.001	< 0.001	0.852
TBARS	1.99	2.37	2.84	1.76	2.17	2.59	1.70	2.02	2.41	0.18	0.004	< 0.001	0.982

Values, expressed as μmol/L, are means with pooled SEM, *n* = 10 sows/treatment group

Data were analyzed by two-way ANOVA

^1^Treatment (T), Day (D), Treatment × Day (T × D) interaction; ^2^cysteine + 1/2 cystine

**Table 8:** Effects of supplementing branched-chain amino acids (BCAAs) to the diets of sows on the concentrations of free amino acids and thiobarbituric acid reactive substances (TBARS) in the plasma of sow-reared piglets during lactation

Variable	Control			1.535% BCAA			3.07% BCAA			Pooled SEM	***P***-value		
	d 3	d 15	d 29	d 3	d 15	d 29	d 3	d 15	d 29		Treatment	Day	T × D^**1**^
Ala	636	649	649	760	687	685	764	692	702	24	< 0.001	0.002	0.036
Arg	216	136	127	238	171	173	272	179	183	12	< 0.001	< 0.001	0.881
Asn	78	89	101	106	110	117	119	120	124	4.8	< 0.001	< 0.001	0.132
Asp	14	19	20	18	21	22	19	23	25	1.2	< 0.001	< 0.001	0.754
β-Ala	26	26	27	28	28	26	28	26	27	1.0	0.504	0.336	0.011
Cit	132	76	81	164	82	90	178	107	109	5.4	< 0.001	< 0.001	0.149
Cys^2^	166	165	158	166	169	162	172	171	162	7.1	0.510	0.130	0.973
Gln	709	542	538	748	605	602	809	646	688	32	< 0.001	< 0.001	0.190
Glu	83	83	85	101	107	103	112	116	118	10	< 0.001	0.240	0.906
Gly	1080	1091	1093	1152	1047	1060	1060	1160	1188	32	< 0.001	0.647	0.968
His	98	95	108	97	95	112	97	95	114	4.6	0.854	< 0.001	0.885
Ile	126	128	127	169	179	188	185	198	201	11	< 0.001	0.132	0.732
Leu	198	178	186	223	227	233	240	242	247	18	< 0.001	0.674	0.596
Lys	202	234	232	215	251	243	220	251	242	15	0.206	0.001	0.990
Met	78	80	77	83	85	79	86	89	91	3.4	< 0.001	0.413	0.485
Orn	136	95	109	141	103	110	148	98	113	6.1	0.193	< 0.001	0.613
Phe	93	93	96	95	96	98	96	96	97	3.1	0.014	0.794	0.022
Pro	500	509	510	526	534	540	541	568	573	14	< 0.001	0.060	0.783
Ser	251	294	301	269	294	299	269	291	302	12	0.703	< 0.001	0.617
Tau	113	128	123	141	137	157	143	170	167	8.0	< 0.001	0.001	0.039
Thr	261	241	243	273	249	253	296	260	269	14	0.004	< 0.001	0.962
Trp	42	46	48	43	48	59	44	51	59	2.2	0.029	< 0.001	< 0.001
Tyr	128	136	135	141	139	136	146	143	140	6.8	< 0.001	0.532	0.002
Val	226	232	231	321	329	330	351	349	352	16	< 0.001	0.787	0.547
TBARS	4.90	4.06	3.61	5.03	3.96	3.04	5.11	4.15	3.14	0.48	0.804	< 0.001	0.829

Values, expressed as μmol/L, are means with pooled SEM, *n* = 20 piglets/treatment group

Data were analyzed by two-way ANOVA.

^1^Treatment (T), Day (D), and Treatment × Day (T × D) interaction; ^2^cysteine + 1/2 cystine

### Concentrations of free and protein-bound AAs in milk

Concentrations of most free AAs, including Ala, Arg, Asp, Asn, Cit, Glu, Gln, BCAAs and Pro, were greater (*P* < 0.05) in milk from BCAA-supplemented, as compared with the control sows (Table 9). Except for Ala and Trp, the abundance of all free AAs was greatest in milk from sows in the 3.07% BCAA group (Table 9). There was a significant effect (*P* < 0.05) of day on concentrations of all free AAs in milk, but a treatment × day interaction effect was detected only for Gln and Ser (Table 9). Concentrations of all protein-bound AAs were greater (*P* < 0.01) in milk of BCAA-supplemented sows, compared with control sows (Table 10). Concentrations of all protein-bound AAs were affected by day of lactation (*P* < 0.05). There was no treatment × day effect (*P* > 0.05) for any AA in milk protein (Table 10).

**Table 9:** Effects of supplementing branched-chain amino acids (BCAAs) to the diets of sows on the composition of free amino acids in their milk during lactation

Variable	Control			1.535% BCAA			3.07% BCAA			Pooled SEM	***P***-value		
	d 3	d 15	d 29	d 3	d 15	d 29	d 3	d 15	d 29		Treatment	Day	T × D^**1**^
Ala	235	511	635	279	597	730	301	609	663	50	0.025	< 0.01	0.682
Arg	36	64	72	52	73	91	54	87	109	6.7	< 0.01	< 0.01	0.220
Asn	29	108	227	46	132	259	51	141	267	11	< 0.01	< 0.01	0.829
Asp	158	462	480	192	526	593	313	546	639	30	< 0.01	< 0.01	0.075
β-Ala	15	30	41	15	30	45	15	28	41	2.6	0.534	< 0.01	0.634
Cit	6.8	38	46	18	49	68	31	59	78	4.7	< 0.01	< 0.01	0.302
Cys^2^	116	192	362	120	199	358	123	204	370	19	0.706	< 0.01	0.994
Gln	326	1060	3517	439	1170	4025	472	1210	4196	112	< 0.01	< 0.01	0.024
Glu	366	1010	991	419	1140	1070	447	1200	1190	43	< 0.01	< 0.01	0.261
Gly	294	812	1180	307	830	1240	358	843	1300	57	0.096	< 0.01	0.844
His	787	682	436	769	689	465	824	688	486	35	0.269	< 0.01	0.737
Ile	5.2	17	21	14	31	38	29	43	52	3.7	< 0.01	< 0.01	0.546
Leu	23	43	51	52	77	93	64	85	103	8.1	< 0.01	< 0.01	0.772
Lys	32	52	68	35	66	86	50	66	85	4.5	< 0.01	< 0.01	0.063
Met	2.9	20	23	4.2	21	28	4.2	22	28	2.2	0.059	< 0.01	0.448
Orn	29	45	64	30	48	67	30	52	58	3.9	0.548	< 0.01	0.178
Phe	20	35	38	25	36	39	27	38	40	2.8	0.040	< 0.01	0.776
Pro	34	82	114	46	115	147	49	123	168	12	< 0.01	< 0.01	0.195
Ser	45	221	403	51	231	474	63	238	381	18	0.012	< 0.01	< 0.001
Tau	1020	1280	1410	1110	1290	1430	1230	1290	1490	99	0.212	< 0.01	0.722
Thr	86	133	426	89	140	437	85	145	420	23	0.862	< 0.01	0.962
Trp	3.6	11	17	5.3	12	21	5.7	12	19	1.1	0.012	< 0.01	0.378
Tyr	35	63	75	42	63	76	49	66	79	5.7	0.114	< 0.01	0.690
Val	54	108	134	96	132	160	149	166	229	13	< 0.01	< 0.01	0.150

Values, expressed as μmol/L of whole milk, are means with pooled SEM, *n* = 10 sows/treatment group

Data were analyzed by two-way ANOVA

^1^Treatment (T), Day (D), and Treatment × Day (T × D) interaction; ^2^cysteine + 1/2 cystine

**Table 10:** Effects of supplementing branched-chain amino acids (BCAAs) to the diets of sows on the composition of protein-bound amino acids in their milk during lactation

Variable	Control			1.535% BCAA			3.07% BCAA			Pooled SEM	***P***-value		
	d 3	d 15	d 29	d 3	d 15	d 29	d 3	d 15	d 29		Treatment	Day	T × D^**1**^
Ala	27.8	15.2	14.6	29.0	20.1	18.4	29.7	19.6	18.8	1.9	0.015	< 0.001	0.690
Arg	15.0	7.48	7.30	17.0	13.7	13.1	19.6	16.4	15.5	1.5	< 0.001	< 0.001	0.961
Asp + Asn	33.8	20.0	19.2	36.1	26.5	22.4	37.3	30.8	27.2	2.1	< 0.001	< 0.001	0.159
Cys	6.82	3.22	3.08	7.02	3.48	3.35	7.56	3.89	3.73	0.50	0.030	< 0.001	0.975
Glu + Gln	76.2	43.7	41.1	81.7	49.2	47.1	86.9	55.8	54.7	4.6	< 0.001	< 0.001	0.353
Gly	19.3	9.48	9.21	20.7	9.85	9.64	21.5	10.2	9.73	1.2	< 0.001	< 0.001	0.892
His	9.09	5.33	5.26	10.5	5.67	5.48	11.2	5.84	5.66	0.78	< 0.001	< 0.001	0.937
Ile	19.6	11.8	11.0	21.1	13.0	12.6	23.6	14.8	13.9	1.8	< 0.001	< 0.001	0.975
Leu	42.7	26.5	25.3	44.8	26.0	25.2	46.3	27.3	26.8	3.1	< 0.001	< 0.001	0.876
Lys	36.3	24.1	23.2	39.5	26.6	24.8	40.7	28.1	25.1	2.3	< 0.001	< 0.001	1.00
Met	9.90	6.18	5.97	10.6	6.77	6.34	11.3	6.98	6.56	0.70	< 0.001	< 0.001	0.413
Phe	15.8	9.83	9.45	16.7	10.4	10.1	17.2	11.5	10.8	1.1	< 0.001	< 0.001	0.827
Pro	42.1	32.8	31.2	44.6	35.6	34.5	46.8	37.2	36.0	3.7	< 0.001	0.001	0.998
Ser	31.6	16.8	15.3	33.0	18.2	16.1	33.8	19.7	18.3	2.5	< 0.001	< 0.001	0.947
Thr	24.6	14.1	12.7	26.1	14.9	13.7	27.3	15.6	14.1	1.9	< 0.001	< 0.001	0.945
Trp	6.01	3.32	3.18	6.57	3.58	3.39	6.65	3.79	3.54	0.26	< 0.010	< 0.001	0.702
Tyr	18.7	11.9	10.6	19.2	12.4	11.4	20.7	13.5	12.0	1.5	< 0.001	< 0.001	0.946
Val	27.8	15.2	14.4	30.3	16.8	16.0	32.5	17.4	16.1	2.1	< 0.001	< 0.001	0.296

Values, expressed as mmol/L of whole milk, are means with pooled SEM, *n* = 10 sows/treatment group

Data were analyzed by two-way ANOVA

^1^Treatment (T), Day (D), and Treatment × Day (T × D) interaction

### Metabolites in the plasma of sows and piglets and in milk

Effects of BCAA supplementation on concentrations of ammonia, urea, glucose, lactate and pyruvate in the plasma of sows and piglets and in sow’s milk are summarized in Table 11. Concentrations of glucose were greater (*P* < 0.05) in the plasma from BCAA-supplemented sows (*P* = 0.018) and from piglets nursed by those sows (*P* < 0.001) than sows and piglets in the control group. No differences in concentrations of glucose in milk were detected among the three groups of sows (*P* > 0.05). BCAA supplementation did not affect (*P* > 0.05) concentrations of ammonia, urea, lactate or pyruvate either in the plasma of sows and piglets or in sow’s milk. Concentrations of lactate in plasma and milk from sows increased (*P* < 0.05) as lactation advanced, but there were no changes (*P* > 0.05) in other measured metabolites. No treatment × day of lactation interaction had an effect on concentrations of ammonia, urea, lactate or pyruvate in plasma from sows and piglets or in milk from d 3, 15 or 29 of lactation (*P* > 0.05). Acetoacetate and β-hydroxybutyrate were not detected in the ×2.5-diluted plasma of sows and piglets or in milk on the same days of lactation (detection limit = 25 μmol/L).

**Table 11:** Effects of supplementing branched-chain amino acids (BCAAs) to the diets of sows on the concentrations of metabolites in the plasma of sows and piglets and in milk during lactation

Variable	Control			1.535% BCAA			3.07% BCAA			Pooled SEM	***P***-value		
	d 3	d 15	d 29	d 3	d 15	d 29	d 3	d 15	d 29		Treatment	Day	T × D^**1**^
Sow’s plasma													
Ammonia, μmol/L	60	62	62	61	64	65	64	67	68	6.4	0.372	0.665	1.00
Urea, mmol/L	2.88	2.97	3.07	2.74	2.83	2.94	2.59	2.68	2.80	0.28	0.224	0.487	1.00
Glucose, mmol/L	5.38	5.41	5.53	5.47	5.51	5.55	5.59	5.64	5.69	0.12	0.018	0.261	0.99
Lactate, mmol/L	1.27	1.49	1.69	1.28	1.52	1.76	1.25	147	1.66	0.21	0.979	< 0.001	1.00
Pyruvate, μmol/L	135	148	161	136	146	166	134	148	170	14	0.963	< 0.001	0.987
Piglet’s plasma													
Ammonia, μmol/L	64	65	65	66	65	67	66	68	69	2.6	0.173	0.607	1.00
Urea, mmol/L	2.37	2.57	2.79	2.36	2.45	2.55	2.33	2.42	2.51	0.25	0.533	0.200	0.964
Glucose, mmol/L	5.32	5.34	5.44	5.41	5.64	5.73	5.65	5.84	5.84	0.13	< 0.001	0.015	0.723
Lactate, mmol/L	2.37	2.38	2.4	2.44	2.44	2.45	2.52	2.49	2.51	0.15	0.367	0.987	1.00
Pyruvate, μmol/L	154	155	156	157	157	157	158	159	160	10	0.807	0.976	1.00
Sow’s milk													
Ammonia, mmol/L	1.59	1.64	1.66	1.65	1.71	1.72	1.70	1.78	1.81	0.17	0.383	0.677	1.00
Urea, mmol/L	7.23	4.79	5.26	7.04	4.65	5.09	6.92	4.67	5.14	0.58	0.832	< 0.001	0.997
Glucose, mmol/L	0.54	0.589	0.592	0.577	0.62	0.629	0.574	0.633	0.64	0.06	0.384	0.163	1.00
Lactate, mmol/L	0.142	0.17	0.195	0.136	0.167	0.192	0.147	0.175	0.199	0.03	0.909	0.026	1.00
Pyruvate, μmol/L	65	67	73	67	65	74	62	69	79	9.4	0.937	0.111	0.932

Values are means with pooled SEM, *n* = 10 sows/treatment group for plasma and milk, *n* = 20 piglets/treatment group

Data were analyzed by two-way ANOVA

^1^Treatment (T), Day (D), and Treatment × Day (T × D) interaction

### Concentrations of TBARS in the plasma of sows and piglets

Concentrations of TBARS in the plasma of sows increased (*P* < 0.001) as lactation progressed (Table 7), but the opposite changes were detected in plasma from piglets (Table 8). BCAA supplementation reduced (*P* = 0.004) the concentrations of TBARS in plasma from sows compared with control sows (Table 7). Concentrations of TBARS in plasma from piglets did not differ (*P* > 0.05) among the three treatment groups (Table 8).

## Discussion

The mammary gland undergoes metabolic and histological changes during lactation [42, 43]. During lactation, sows are capable of utilizing nutrients from the arterial circulation for milk production [44, 45]. However, due to an increased metabolic rate and a reduced feed intake of lactating sows, they cannot meet the nutritional requirements for milk production, thereby leading to a catabolic state in which their body reserves are mobilized to provide precursors and energy for milk production [1]. This results in a substantial weight loss for lactating sows, which can negatively affect their subsequent reproductive performance [46]. In the present study, we supplemented the diet of lactating sows (fed a conventional diet) with a mixture of L-leucine, L-isoleucine and L-valine in proportion to their physiological ratios in the lumen of their small intestine so as to prevent an imbalance among BCAAs. Results of the present study demonstrated that supplementing 1.535% and 3.07% BCAAs to the typical corn- and soybean meal-based diet of multiparous sows during the entire 29-d lactation period reduced their weight loss, while increasing milk yield as well as concentrations of total AAs, lipids and lactose in milk, and also improved piglet growth performance and survival. Therefore, findings of the present study provide a new and effective strategy for nutritional management of lactating sows. To our knowledge, this is the first study demonstrating effects of dietary supplementation with a mixture of BCAAs (without increasing dietary protein levels) on the lactational performance of lactating sows and associated effects on the enhanced growth and reduced mortality of preweaning piglets, as compared to an isonitrogenous control.

Lactating mammals extensively degrade BCAAs [47, 48]; therefore, there are increased requirements for dietary BCAAs by lactating sows to support their milk production [49]. There are reports that supplementing L-valine to the corn- and soybean-based diets of lactating sows enhanced milk yield and piglet growth. For example, Richert et al. [15] found that litter weaning weight and litter weight gain in high-producing lactating sows increased as the total content of valine and isoleucine in the diet increased from 0.72% to 1.42% and from 0.50% to 1.2%, respectively. Also, increasing total valine content from 0.61% to 1.15% in the diet for lactating sows nursing 10 or more piglets increased litter preweaning weights [18]. This beneficial effect of dietary valine (an augmentation from 0.75% to 1.15% valine) on lactation was confirmed by Moser et al. [16]. Furthermore, Paulicks et al. [17] observed that supplementing up to 1% valine to a corn- and soybean meal-based diet containing 0.45% valine for lactating sows increased milk production and total milk protein content, in comparison with the control group. 

Research with lactating sows and cows has shown that effects of dietary supplementation with leucine or valine on milk yield and composition depend on the doses of the supplemental AA and the balance of dietary BCAAs. For example, supplementing either 0.4% leucine to a corn- and soybean-based diet containing 1.57% leucine or 0.4% isoleucine to a basal diet containing 0.68% isoleucine for lactating sows did not affect their milk production or piglet growth [16]. Results of other studies also indicated that the lactation performance of sows was not affected by supplementing (a) 0.26–0.37% valine to a corn- and soybean meal-based diet containing 0.61% valine, 0.58% isoleucine, and 0.95% leucine [15]; (b) 0.4% isoleucine to the basal diet containing 0.68% isoleucine, 0.8% valine, and 1.57% leucine [18]; (c) 0.4% leucine to the basal diet containing 1.57% leucine, 0.8% valine, and 0.68% isoleucine [18]; or (d) by increasing the total dietary valine:lysine ratio from 0.84 to 0.91 [24]. Interestingly, the duodenal infusion of 40 g/d leucine to lactating cows increased concentrations of casein, whey proteins, and total proteins in milk, compared with 0 g/d leucine [50]. However, the duodenal infusion of 40, 80 and 120 g/d leucine to lactating cows dose-dependently reduced milk fat and lactose yields in comparison with 0 g/d leucine, whereas the duodenal infusion of 80 g/d leucine decreased the total protein yield and the true-protein content of milk compared with 40 g/d leucine [50]. Because BCAAs share the same transmembrane transporters and the initial enzymes for catabolism [51], ratios of leucine, isoleucine and valine, as well as the content of other AAs and nutrients in the complete diet may affect lactational performance. To prevent a BCAA imbalance resulting from the addition of one single BCAA to the ration, dietary supplementation with all the three BCAAs in proper ratios provides a more effective strategy to improve milk production in sows and perhaps other mammals.

Voluntary feed intake of sows was not affected by dietary supplementation with up to 3.07% BCAAs (Table 3). As reported previously [52], the body weight of sows was markedly reduced with advanced lactation (Table 4). Interestingly, dietary supplementation with 1.535% BCAAs and 3.07% BCAAs reduced the weight loss of sows in a dose-dependent manner (Table 4), which may be attributed to effects of BCAAs (mainly leucine and valine) to stimulate muscle protein synthesis and inhibit muscle proteolysis during catabolic conditions [28, 51, 53], and those effects may enhance the subsequent reproductive performance of sows. In the present study, we did not measure the component of weight loss (e.g., protein, fats, and/or minerals) in lactating sows, but the loss likely occurred primarily in white adipose tissue and secondarily in skeletal muscle [52]. It is likely that by activating the MTOR cell signaling pathway, dietary BCAA supplementation improves the efficiency of the utilization of dietary nutrients for milk production.

Approximately 40% to 45% of dietary BCAAs are utilized by the small intestine of sows in the first pass [23]. Because the degradation of BCAAs in the liver is limited due to its low BCAA transaminase activity [22], most of the BCAAs that enter the portal circulation bypasses this organ to become available for utilization by other tissues, including skeletal muscle and mammary glands [11, 54]. Skeletal muscle of lactating mammals actively transports extracellular BCAAs and converts them into BCKAs and glutamate [22]. Glutamate is subsequently amidated with NH_4_^+^ to form glutamine and undergoes transamination with pyruvate and oxaloacetate to yield alanine and aspartate, respectively [22, 51]. Aspartate is then amidated by asparagine synthetase (an ATP- and glutamine-dependent cytosolic enzyme) to form asparagine [51]. This explains why dietary BCAA supplementation increased concentrations of glutamate, glutamine, alanine, and asparagine in the plasma of lactating sows. Utilization of arterial glutamine by the small intestine of lactating sows is expected to promote the intestinal synthesis of citrulline (the precursor of arginine), thereby contributing to an increase in the concentrations of both citrulline and arginine in maternal blood (Table 7).

Besides skeletal muscle and small intestine, the mammary gland of sows takes up and catabolizes large amounts of extracellular BCAAs [22, 55]. For example, during lactation, 76–80 g/d BCAAs are utilized by the mammary gland, with 46–53 g/d BCAAs being secreted in milk [56, 57]. Thus, approximately 30 g/d BCAAs are metabolized in the mammary gland. We reported that BCAAs are nitrogenous precursors for the synthesis of glutamate, glutamine, aspartate, asparagine, and alanine in the lactating mammary gland, which contains BCAA transaminase, branched-chain α-ketoacids (BCKA) dehydrogenase, glutamine synthetase, glutamate-oxaloacetate aminotransferase, glutamate-pyruvate aminotransferase, and asparagine synthetase [22, 58]. Similarly, Matsumoto et al. [59] found that dietary supplementation with leucine increased concentrations of glutamate and glutamine in the milk of rats. The BCKAs produced can be converted into acetyl-CoA, which can be either oxidized to provide ATP or utilized for fat synthesis. Of note, dietary supplementation with 2 g/d β-hydroxy-β-methyl butyrate (a leucine metabolite) to sows enhances fat content in colostrum by 41% and the body weight of suckling pigs by 7% on d 21 of lactation [60]. Taken together, these results help to explain our observation that dietary supplementation with BCAAs increased fat concentration in milk even when lactating sows were mobilizing less from body reserves. Furthermore, BCAAs stimulate protein synthesis in mammary epithelial cells through the MTOR pathway [27]. The results of the present study advance our understanding of lactation biology. These new and important findings also reveal that dietary BCAA supplementation increases concentrations of free and peptide-bound glutamate, glutamine, aspartate, asparagine, and alanine, as well as concentrations of protein and fat in sow’s milk.

As noted previously, an inadequate provision of dietary protein and some AAs (e.g., arginine, glutamine, glutamate, glycine, leucine, and proline) limits the maximal growth of young pigs [11, 23, 61, 62]. An increase in the provision of these AAs from sow’s milk enhances their concentrations in the plasma of suckling piglets, thereby improving the growth, muscular strength, intestinal health, immunity, and survival of suckling piglets. The underlying molecular mechanisms may involve the supply of substrates for protein synthesis as well as activation of the MTOR cell signaling pathway to increase protein synthesis and inhibit protein degradation [25, 63–65]. In support of this view, arginine and glycine are known to be severely deficient in sow’s milk relative to their requirements for piglet growth [19, 20, 66]. Additionally, approximately 70% and 95% of glutamine and glutamate in sow’s milk are utilized via catabolic and synthetic pathways in the small intestine of piglets during the first-pass metabolism and, therefore, do not enter the portal circulation [67, 68]. Thus, the endogenous synthesis of these two AAs, which are highly abundant in tissue protein, is necessary for piglet growth [23, 69]. Furthermore, arginine, glutamine, glycine, and leucine stimulate the phosphorylation of MTOR, S6K1, and 4EBP1 proteins in porcine tissues, including mammary tissue [64], skeletal muscle [26, 54, 70], and the small intestine [62, 71]. In addition, valine has recently been reported to activate the MTOR cell signaling to promote milk protein synthesis in porcine mammary epithelial cells [28]. These AAs can also enhance the expression of antioxidative genes and reduce the expression of proinflammatory genes in tissues [51, 72], thereby attenuating oxidative stress in swine (as indicated by the reduced concentration of TBARS in the plasma; Table 7) and improving their health and lactation performance. Similarly, dietary supplementation with BCAAs reduced oxidative stress in both rats with liver cirrhosis [73] and exercising humans [74]. Taken together, these functional AAs are expected to play an important role in improving the growth of neonatal pigs and the efficiency of milk utilization [75–78].

## Conclusions

Supplementing a proper mixture of BCAAs to the diet for lactating sows increases concentrations of BCAAs, glutamate, glutamine, citrulline, arginine, proline, asparagine, and many other AAs in the plasma and milk of sows, and the plasma of piglets, while reducing oxidative stress in sows and increasing plasma glucose concentrations in both sows and piglets. BCAA supplementation does not affect either feed intake by sows or milk consumption by piglets (per kg BW), but enhances the growth of suckling piglets likely through stimulating tissue protein synthesis. Dietary supplementation with appropriate proportions of leucine, isoleucine and valine can prevent an imbalance among BCAAs in the diet and provide an effective means to improve milk synthesis by lactating sows and, in turn, the growth and survival of piglets.

## Data Availability

All data generated or analyzed during this study are available from the corresponding author upon reasonable request.

## Abbreviations

AAs: Amino acids
β-Ala: Beta-alanine
BCAAs: Branched-chain amino acids
BCKAs: Branched-chain α-ketoacids
Cit: Citrulline
CP: Crude protein
DM: Dry matter
MTOR: Mechanistic target of rapamycin
Orn: Ornithine
TAU: Taurine
TBARS: Thiobarbituric acid reactive substances
